# Supplementary material for: Event-related potentials of stimuli inhibition and access in cross-modal distractor-induced blindness
Source: PLoS One. 2024 Oct 23;19(10):e0309425. doi: 10.1371/journal.pone.0309425 (PMC11498723; doi:10.1371/journal.pone.0309425)

S3 Fig

Graphical representation of the mean amplitudes for the distractor-evoked ERP following both liberal and conservative artifact rejection, categorized by distractor condition.

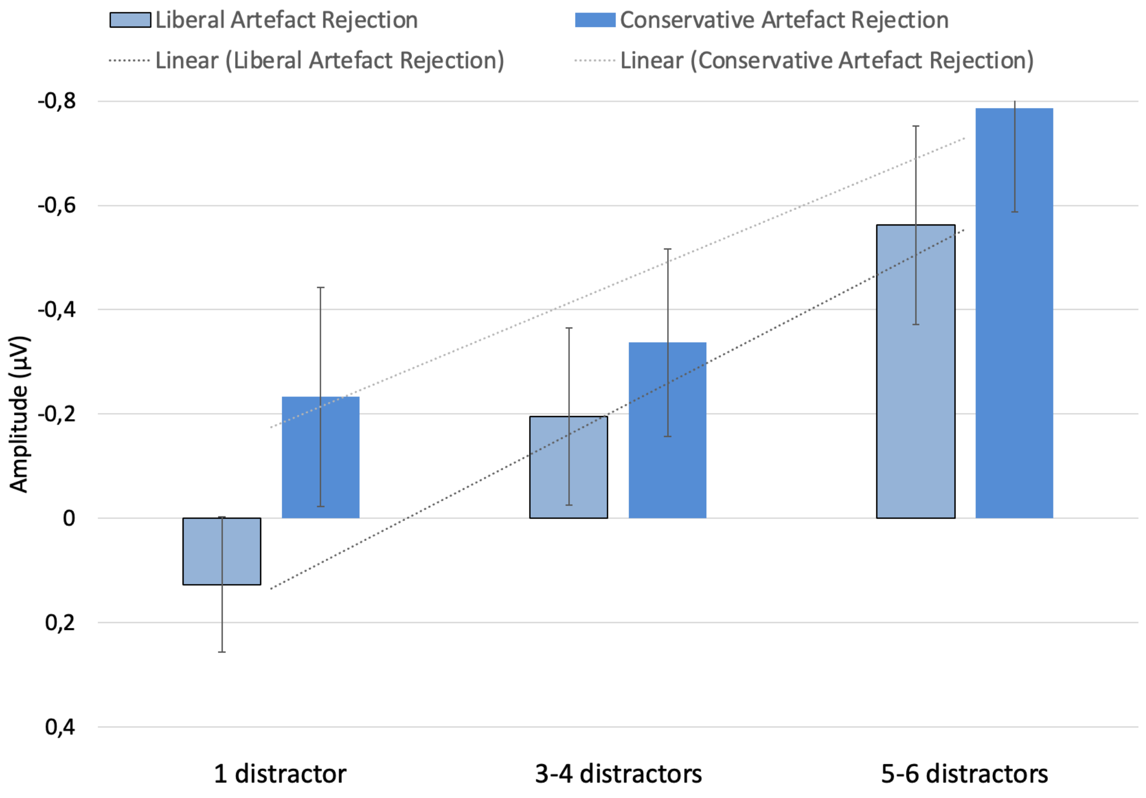

Supplement: S1 Fig — (PDF) [file pone.0309425.s001.pdf]
