# Supplementary material for: Event-related potentials of stimuli inhibition and access in cross-modal distractor-induced blindness
Source: PLoS One. 2024 Oct 23;19(10):e0309425. doi: 10.1371/journal.pone.0309425 (PMC11498723; doi:10.1371/journal.pone.0309425)
Supplement: S1 Table — (PDF) [file pone.0309425.s002.pdf]

S1 Table

Mean target detection rates for the pilot as well as for the present study, based on 20 trials per experimental condition.

Distractors	Pilot study (SOA 0 ms)	Present study (SOA 0 ms)
0	M = 90.45<br>CI [83.81, 97.08]	M = 94.74<br>CI [91.05, 98.44]
1	M = 87.16<br>CI [79.48, 94.84]	M = 88.36<br>CI [83.41, 93.31]
3-4	M = 75.81<br>CI [65.41, 86.20]	M = 82.37<br>CI [75.64, 89,10]
5-6	M = 70.25<br>CI [56.63, 83.86]	M = 74,37<br>CI [66.15, 82.59]

Note. Mean (in %) and the corresponding 95% confidence interval (CI) for averaged correct target detection after correct cue detection, reported separately for each distractor condition. SOA = stimulus onset asynchrony.

Both experiments, the pilot study as well as the present ERP study, clearly indicate that the hits rate is affected significantly by the number of distractors. For the pilot study, the effect of the number of distractors was strongly expressed ($F(1.76, 24.58) = 10.90$, $p <.001$, $n_p^2 = .438$) and followed a linear trend ($F(1,14) = 17.48$, $p < .001$, $n_p^2 = .555$).

What is more, a combined analysis (mixed ANOVA) of both experiments highlights the reliability of the observed behavioral effect: No significant interaction between the within-subjects factor ‘distractor number’ (0 vs. 1 vs. 3-4 vs. 5-6) and the between-subjects factor ‘experiment’ (pilot vs. present experiment) could be found ($F(3,120) = .44$, $p < .726$, $n_p^2 = .011$).
