# Supplementary material for: Event-related potentials of stimuli inhibition and access in cross-modal distractor-induced blindness
Source: PLoS One. 2024 Oct 23;19(10):e0309425. doi: 10.1371/journal.pone.0309425 (PMC11498723; doi:10.1371/journal.pone.0309425)
Supplement: S2 Table — (PDF) [file pone.0309425.s003.pdf]

S2 Table

Mean amplitudes for the distractor-evoked ERP after the liberal and conservative artefact rejection per distractor condition, including the corresponding number of trials they were based on.

Condition	Frontal Negativity (90-170 ms)			
	Liberal Artefact Rejection (24% rejection rate)		Conservative Artefact Rejection (52% rejection rate)	
1 distractor	M = .127 [-.14, .393]	GA based on M = 42.96 trials (SD = 9.97)	M = -.233 [-.66, .20]	GA based on M = 27.15 trials (SD = 6.73)
3-4 distractors	M = -.195 [-.551, .161]	GA based on M = 41.48 trials (SD = 10.61)	M = -.337 [-.70, .03]	GA based on M = 27.00 trials (SD = 6.55)
5-6 distractors	M = -.562 [-9.49, -.18]	GA based on M = 41.89 trials (SD = 9.11)	M = -.787 [-1.19, -.39]	GA based on M = 25.19 trials (SD = 7.55)

Note. Mean (in μV) and the corresponding 95% confidence interval (CI) for the grand averaged (GA) waveforms. Number of trials remaining after each artifact rejection process (liberal vs. conservative) is indicated by the mean and corresponding standard deviation. While the liberal rejection process lead to a rejection rate of 24%, the conservative rejection lead to a rejection rate of 52%.

Our analysis showed that the reliability of the frontal ERP signal was still provided after the liberal artefact rejection: A cross-correlation of the averages per participant resulted in a mean correlation coefficient of .68 (SD = .18) for the 'single distractor' condition, .69 (SD = .19) for the '3-4 distractors' condition, and .69 (SD = .18) for the '5-6 distractors' condition. A statistical analysis of the new ERP signals at the frontal leads in the time range of interest (90-170 ms) affirmed an increase in negativity (see also S3 Figure). More importantly, the rmANOVA also confirmed a significant linear trend ($F(1,26) = 8.11$, $p = .008$, $n_p^2 = .238$), consistent with the previously analyzed data after the conservative artefact rejection.
