# Supplementary material for: Event-related potentials of stimuli inhibition and access in cross-modal distractor-induced blindness
Source: PLoS One. 2024 Oct 23;19(10):e0309425. doi: 10.1371/journal.pone.0309425 (PMC11498723; doi:10.1371/journal.pone.0309425)
Supplement: S3 Table — (PDF) [file pone.0309425.s004.pdf]

S4 Table

Post hoc paired t-tests for the five electrodes of the parietal cluster, comparing the conditions 'cue-target' vs. 'cue-only'.

Electrodes	Difference of the means (M)	Standard deviation (SD)	T-value	Degrees of freedom (df)	One-tailed p-value (p)	Effect size (Cohen's d)
**P3**	.209	2.80	.39	26	.350	2.797
**P4**	.801	3.24	1.29	26	.105	3.238
**Pz**	.012	3.20	.02	26	.493	3.195
**CP1**	-.608	3.03	-1.05	26	.153	3.026
**CP2**	-.351	3.15	-.58	26	.284	3.152
