# Supplementary material for: Event-related potentials of stimuli inhibition and access in cross-modal distractor-induced blindness
Source: PLoS One. 2024 Oct 23;19(10):e0309425. doi: 10.1371/journal.pone.0309425 (PMC11498723; doi:10.1371/journal.pone.0309425)
Supplement: S4 Table — (PDF) [file pone.0309425.s005.pdf]

S5 Table

Post hoc paired t-tests for the five electrodes of the parietal cluster, comparing the conditions ‘low’ vs. ‘high’ distractor number.

**Electrodes**	**Difference of the means (M)**	**Standard deviation (SD)**	**T-value**	**Degrees of freedom (df)**	**One-tailed p-value (p)**	**Effect size (Cohen’s d)**
**P3**	-1.328	2.79	-2.47	26	.010	2.789
**P4**	-1.174	3.09	-1.98	26	.029	3.089
**Pz**	-1.371	3.63	-1.96	26	.030	3.632
**CP1**	-.536	3.10	-.90	26	.188	3.095
**CP2**	-.776	3.15	-1.28	26	.106	3.150
