# Supplementary material for: Event-related potentials of stimuli inhibition and access in cross-modal distractor-induced blindness
Source: PLoS One. 2024 Oct 23;19(10):e0309425. doi: 10.1371/journal.pone.0309425 (PMC11498723; doi:10.1371/journal.pone.0309425)
Supplement: S5 Table — (PDF) [file pone.0309425.s006.pdf]

S6 Table

Post hoc paired t-tests for the five electrodes of the posterior cluster, comparing the conditions 'cue-target' vs. 'cue-only'.

Electrodes	Difference of the means (M)	Standard deviation (SD)	T-value	Degrees of freedom (df)	One-tailed p-value (p)	Effect size (Cohen's d)
**O1**	-2.336	2.69	-4.51	26	<.001	2.693
**O2**	-2.549	2.59	-5.11	26	<.001	2.593
**Oz**	-1.811	2.04	-4.61	26	<.001	2.040
**P7**	-1.556	2.27	-3.56	26	<.001	2.268
**P8**	-1.727	2.34	-3.84	26	<.001	2.335
