# Supplementary material for: Event-related potentials of stimuli inhibition and access in cross-modal distractor-induced blindness
Source: PLoS One. 2024 Oct 23;19(10):e0309425. doi: 10.1371/journal.pone.0309425 (PMC11498723; doi:10.1371/journal.pone.0309425)
Supplement: S6 Table — (PDF) [file pone.0309425.s007.pdf]

S7 Table

Post hoc paired t-tests for the five electrodes of the posterior cluster, comparing the conditions ‘low’ vs. ‘high’ distractor number.

Electrodes	Difference of the means (M)	Standard deviation (SD)	T-value	Degrees of freedom (df)	One-tailed p-value (p)	Effect size (Cohen’s d)
**O1**	-1.562	2.15	-3.77	26	<.001	2.153
**O2**	-1.615	2.30	-3.65	26	<.001	2.299
**Oz**	-.850	1.72	-2.57	26	.008	1.717
**P7**	-1.767	1.65	-5.57	26	<.001	1.647
**P8**	-1.874	2.12	-4.59	26	<.001	2.122
