# Supplementary material for: Event-related potentials of stimuli inhibition and access in cross-modal distractor-induced blindness
Source: PLoS One. 2024 Oct 23;19(10):e0309425. doi: 10.1371/journal.pone.0309425 (PMC11498723; doi:10.1371/journal.pone.0309425)
Supplement: Attachment — Submitted filename: Response to Reviewers.docx [file pone.0309425.s008.docx]

Dear Dr. Patrick Bruns,

Please find enclosed our revised manuscript (Event-related potentials of stimuli inhibition and access in cross-modal distractor-induced blindness) submitted for your consideration for publication in PLOS ONE.

First and foremost, we would like to extend our gratitude to you and the reviewers for your insightful comments. We have carefully considered all feedback, which has significantly enhanced the quality and comprehensibility of the manuscript.

In response to the reviewers' points, we have thoroughly addressed each one: Specifically, we have elaborated on the relationship between distractor-induced blindness (DIB) and the conceptually related effect of repetition blindness. Furthermore, we trust that our detailed explanations and the amendments made to the manuscript will convince you and the reviewers that our paradigm can indeed be accurately described as 'cross-modal'.

The corresponding changes in the revised manuscript (see: Revised Manuscript with Track Changes) are detailed below, point by point. We hope that these substantial revisions and additions will meet the reviewers' expectations and that the improved quality of the manuscript will be sufficient for publication in your esteemed journal.

Sincerely,

Sophie Hanke (on behalf of the authors)

**Reviewer #1:**

Thank you very much for your detailed and valuable feedback. Below you will find our responses to your comments. The part in bold font has been incorporated into the revised manuscript.

*The authors are discussing relationships between DIB and attentional blink, and between DIB and contingent attentional capture, but I think that repetition blindness is more relevant here. The reason is that target-distractor similarity is neither a crucial factor for attentional blink nor for contingent-attentional capture, but target-distractor similarity is a crucial factor in repetition blindness. Therefore, I suggest to explain the role of target-distractor similarity for the DIB in more detail, and to discuss the relationship (i.e., similarities and differences) between DIB and repetition blindness.*

Thank you for your comment. We have thoroughly reviewed the phenomenon of repetition blindness (RB; Arnell & Shapiro, 2011; Coltheart, 2010), which refers to the inability to report the second of two targets (either words or pictures) presented in a RSVP stream, if the same target has been presented previously.

After careful consideration, we came to the conclusion that the same difference between DIB and contingent attentional capture, already highlighted in the original manuscript, also accounts for RB: Despite the obvious similarity to the DIB effect concerning the physical resemblance between distractor and target stimuli, previous DIB results revealed that a single distractor is not sufficient to elicit the effect, but that the effect requires the presentation of multiple distractors (Winther & Niedeggen, 2017b). In RB, the simple repetition of the target item is sufficient to elicit the effect. This difference might (also) be attributed to the choice of the physical stimuli: In RB, highly complex semantic stimuli are used, and a token individuation is assumed to be required (Kanwisher, 1987). In DIB, stimuli are very simple visual and/or auditory features which are not to be processed on a semantic level.

We refer to the difference in the paradigms in the revised manuscript:

Page 30: “**RB refers to the inability to report the second occurrence of a word (or picture) presented twice in an RSVP stream (Arnell & Shapiro, 2011; Coltheart, 2010). This discrepancy has been attributed to a lack of token individuation (Kanwisher, 1987), or an ‘offline’ retrieval problem (Whittlesea & Masson, 2005). A crucial factor for RB to occur is the physical resemblance between the two critical stimuli. Likewise in DIB, target processing is impeded solely by the presence of similar distractors, rather than dissimilar or absent ones (Michael et al., 2011). Despite this evident similarity between both phenomena, research indicates that in RB the mere repetition of the target item once is sufficient to induce an effect. As discussed above, such an experimental condition seems not adequate to elicit DIB (Winther & Niedeggen, 2017b). This difference between the two phenomena may be attributed to the distinct choice of physical stimuli: In RB, highly complex semantic stimuli are employed, requiring token individuation. In contrast, DIB involves stimuli comprising basic visual and/or auditory features that do not necessitate semantic-level processing.”**

*Stylistic suggestions:*

- *Page 15, lines 362-363: The sentence „Correspondingly to the definition (…“ seems incomplete.*
- *Page 24, line 565: „stating“ should be „starting“.*

Thank you kindly for highlighting these stylistic aspects that we missed during our initial proofreading. Both amendments have been incorporated into the revised manuscript.

**Reviewer #2:**

Thank you very much for your kind and thoughtful feedback. Please find our detailed responses, to all the points you raised, below. The sections that are written in bold font have been incorporated into the revised manuscript, improving its quality substantially.

*Based on my understanding of the approach, what were manipulated “cross-modal” in the DIB paradigm was actually the cue vs. the target and distractors. While what DIB truly concerns is the effect of target-like distractor exposure (IV) on target identification (DV), I feel that the cue, as a non-critical variable, plays a relatively small role in this effect. Maybe the authors could elaborate on why it is necessary to vary the modality of the cue, and any implications from the cross-modal study above and beyond the uni-modal study of this effect. This would establish the foundation for using EEG to study such an effect in the first place.*

Thank you for your comment. We have to admit that the motivation for the cross-modal approach was not sufficiently elucidated in our original manuscript.

Congruent with the reviewer’s note, previous experiments on the visual DIB indicated that the cue merely serves as a release signal. The effect does not critically depend on the spatial position of the cue (peripheral (Niedeggen et al., 2004) vs. central stream (Milders et al., 2004; Niedeggen et al., 2002; Sahraie et al., 2001)). Moreover, the feature of the cue type does not modulate the effect (letter (Hay et al., 2006) vs. luminance change (Winther & Niedeggen, 2017a, 2018) vs. onset of ‘transparent’ motion (Niedeggen et al., 2004), vs. color change (Milders et al., 2004; Niedeggen et al., 2015; Niedeggen et al., 2012; Niedeggen et al., 2002; Sahraie et al., 2001; Winther & Niedeggen, 2017b). This point has been addressed in previous studies (Hay et al., 2006). Notably, the design remains unimodal with respect to cue and target in all studies.

However, electrophysiological findings already suggested that the processing of the cue-target complex is different when cue and target were defined in distinct modalities (Kern & Niedeggen, 2021b): For unimodal DID (cue and target presented in the auditory domain), ERP responses to the cue-target compound were still characterized by a prominent P3 – if the target is detected successfully (Kern & Niedeggen, 2021a). Yet, correctly reported targets were additionally characterized by a stronger fronto-central negativity at about 200 ms. What is more, for the cross-modal DID effect (visual cue and auditory target), successful target detection was exclusively characterized by a pronounced fronto-central negativity, whereas the P3 amplitude was not enhanced when contrasted to misses (Kern & Niedeggen, 2021b).

These results suggests that the processing of the target is affected by the modality of the cue. From our point of view, this finding qualifies the task used in our study as a ‘cross-modal’ task.

We have clarified this point in our revised paper:

Page 5: “**In sum, these deviant ERP results signal that the central suppression process previously identified in uni-modal visual distractor studies must be questioned if cue and target are defined in different modalities.** **The processing of a target stimulus in a multiple-distractor task seems to be affected by the congruency of the cue’s modality. In this respect, we refer to studies in which cue and target are defined in the same modality as ‘unimodal’. The current study defines the cue and target in different sensory domains and is labeled ‘cross-modal’.”**

Please note that the ERP results of our study also emphasize the role of the different cue/target modalities: The posterior negativity – not observed in the uni-modal visual DIB studies (Niedeggen et al., 2015; Niedeggen et al., 2004; Niedeggen et al., 2012; Winther & Niedeggen, 2017a) – has probably been masked by the choice of the visual cue. Finally, the label ‘cross-modal’ is consistent with the labeling used in the previous studies – based on a similar setup (Kern & Niedeggen, 2021b, 2023).

Page 35: “**Replacing the visual cue by an auditory one reveals the discernibility of the VAN in the context of target processing. This suggests expanding the assumption that the cue merely serves as a temporal marker resolving attentional inhibition (Hay et al., 2006): The congruency of the cue with the target's modality appears to be an important experimental factor. To emphasize this fact, we decided to label the DIB effect associated with an auditory cue as a ‘cross-modal’ setup. Furthermore, this labeling is consistent with the labeling used in previous studies (Kern & Niedeggen, 2021b, 2023).**”

*On Page 11 and 14, the number of trials in each cell condition seems too small to me, both in terms of obtaining a reliable mean behavioral target detection response and reliable averaged ERP waveforms. Although the behavioral results even showed an increase in the DIB effect compared to previous studies, the small number of trials in ERP averaging made it questionable that the several null effects in ERP analysis could be due to relatively large variability without an ample number of trials. The lack of a replication experiment further exacerbates my concern on the interpretation of the null effects. The number of trials used by earlier experiments that successfully detected those ERPs of interest may be cited in support of the current design.*

We appreciate you bringing this important point to our attention and recognize that we did not communicate this information clearly enough in our original manuscript.

As for the ERP analysis, the number of trials available for the analysis of the distractor effect was sufficient: Please note that for each distractor condition (distractor number 1 vs. 3-4 vs. 5-6) a total of 55 trials per condition was foreseen. The ERP analysis of distractor effects in previous studies was based on a comparable number (n=63 trials per distractor condition; Niedeggen et al., 2012; Winther & Niedeggen, 2017a). As in previous experiments, this number also considered a rigorous artefact rejection, leading to the loss of approximately 50% of the trials (Kern & Niedeggen, 2021b: In the distractor-sensitive subgroup, a mean of 50.76 hit trials (SD=12.19) out of 80 trials remained after artifact rejection; In the distractor-insensitive subgroup, a mean of 38.77 hit trials (SD=9.97) out of 80 trials remained after artifact rejection). The rejection rate in the present study was comparable to that of previous studies, leading to an analogous signal-to-noise ratio of the averaged ERPs in single participants.

This point has been clarified in the revised manuscript:

Page 11: “**For the analysis of the distractor-evoked potentials, 55 trials were available for each distractor condition (1 vs. 3-4 vs. 5-6), with the SOA and control conditions combined. In previous studies, the analysis of distractor-evoked ERPs was based on a comparable number (n=63 trials per distractor condition; Niedeggen et al., 2012; Winther & Niedeggen, 2017a).**”

As well as page 14: **“The rejection rate in the present study was comparable to that of a previous experiment (Kern & Niedeggen, 2021b: In the distractor-sensitive subgroup, a mean of 50.76 hit trials (SD = 12.19) out of 80 trials remained after artifact rejection; In the distractor-insensitive subgroup, a mean of 38.77 hit trials (SD = 9.97) out of 80 trials remained after artifact rejection), leading to an analogous signal-to-noise ratio of the averaged ERPs in single participants.”**

As for the behavioral analysis, the computation of the mean hit rate in each participant was based on 20 trials per distractor condition. This trial number was based in a pilot study, and we can take these results to estimate the reliability of the effect.

S1 Table

*Mean target detection rates for the pilot as well as for the present study, based on 20 trials per experimental condition.*

| Distractors | **Pilot study** (n=15) (SOA 0 ms condition) | **Present study** (n=27) (SOA 0 ms condition) |
| --- | --- | --- |
| 0 | M = 90.45 | M = 94.74 |
|  | CI [83.81, 97.08] | CI [91.05, 98.44] |
| 1 | M = 87.16 | M = 88.36 |
|  | CI [79.48, 94.84] | CI [83.41, 93.31] |
| 3-4 | M = 75.81 | M = 82.37 |
|  | CI [65.41, 86.20] | CI [75.64, 89,10] |
| 5-6 | M = 70.25 | M = 74,37 |
|  | CI [56.63, 83.86] | CI [66.15, 82.59] |

*Note.* Mean (in %) and the corresponding 95% confidence interval (CI) for averaged correct target detection after correct cue detection, reported separately for each distractor condition. SOA = stimulus onset asynchrony.

Both experiments, the pilot study as well as the present ERP study, clearly indicate that the hit rate is affected significantly by the number of distractors. For the pilot study, the effect of the distractor number was strongly expressed (F(1.76, 24.58) = 10.90, p <.001, n_p_^2^ = .438) and followed a linear trend (F(1,14) = 17.48, p < .001, n_p_^2^ = .555). Also for the present study (see manuscript page 22), the effect of the distractor number was strongly expressed (F(2.03, 52.89) = 20.92, p <.001, n_p_^2^ = .446) and followed a linear trend (F(1,26) = 32.27, p < .001, n_p_^2^ = .554).

What is more, a combined analysis (mixed ANOVA) of both experiments highlights the reliability of the observed behavioral effect: No significant interaction between the within-subjects factor ‘distractor number’ (0 vs. 1 vs. 3-4 vs. 5-6) and the between-subjects factor ‘experiment’ (pilot vs. present experiment) could be found (F(3,120) = .44, p < .726, n_p_^2^ = .011).

In the revised manuscript, we now included that the estimation of trial number necessary to reveal a distractor effect was based on a pilot study, and that the results of the pilot study can be found in the supplement:

Page 11: “**This number of trials was based on the outcome of a pilot study (n=15 participants), see supplement (S1 Table). Most importantly, the pilot data confirm the reliability of the effect of distractor number.”**

Last but not least, we fully acknowledge the importance of replicating our results. Additionally, we have made every effort to avoid misinterpreting null results throughout the manuscript.

*Some concepts mentioned in results and discussion were not correspondingly introduced in the Methods section, such as “target complexity” and “perceptual load”. It is possible that the authors framed the terms differently in wording the manipulations, which seems lack of consistency for understanding the article.*

Thank you kindly for highlighting these inconsistencies about the wording used in our manuscript. Both terms were used to describe the visual setup defined by a transparent motion which is clearly different from the setup used in the preceding cross-modal study. To enhance clarity and consistency, we restricted ourselves to the term ‘complexity’, and added the following sentences to the Methods section:

Page 13: “**In the current study, a more complex dynamic pattern was used (two superimposed motion planes). This setup has been previously established by Valdes-Sosa and colleagues (Pinilla et al., 2001; Rodríguez & Valdés-Sosa, 2006; Valdes-Sosa et al., 1998) and requires a visual segregation process. We will refer to this difference in visual stimuli configuration as ‘complexity’ in the following.”**

*When reporting the ANOVA results, the degrees of freedom in F-statistics were decimals in several places. This may indicate unequal variances in the data. The authors may want to double check if any assumptions of the statistical test were violated.*

Thank you for this mindful observation. For all rmANOVAs in our study, first of all, a Mauchly test for sphericity has been calculated, and we can confirm that the assumptions of the ANOVA were not violated. The decimal places rather result from our decision to report the degrees of freedom as corrected by the Greenhouse-Geisser criterion (Greenhouse & Geisser, 1959). If more appropriate, we can also report the uncorrected degrees.

Page 17: **“In all analyses of variance, degrees of freedom were corrected according to the Greenhouse-Geisser criterion (Greenhouse & Geisser, 1959), if the assumption of sphericity was violated.”**

**Reviewer #3:**

Thank you very much for taking the time to read our manuscript and share your feedback with us. Below you will find our response, the sections in bold font have been incorporated into the revised manuscript.

I found the fact that the authors see this as an instance of cross-modal DIB somewhat disturbing because in my mind the modality of the cue is not so relevant. So, when I first read about cross-modal DIB I was of course thinking about distractors and targets being presented to different modalities – yet, this is only personal taste, I guess.

Thank you for this comment. This important point has also been addressed by reviewer 2, which furthermore confirms that the motivation for our cross-modal approach was not sufficiently transparent in the original manuscript.

Previous experiments on the visual DIB indicated that the cue merely serves as a release signal. The effect does not critically depend on the spatial position of the cue (peripheral (Niedeggen et al., 2004) vs. central stream (Milders et al., 2004; Niedeggen et al., 2002; Sahraie et al., 2001)). Moreover, the feature of the cue type does not modulate the effect (letter (Hay et al., 2006) vs. luminance change (Winther & Niedeggen, 2017a, 2018) vs. onset of ‘transparent’ motion (Niedeggen et al., 2004), vs. color change (Milders et al., 2004; Niedeggen et al., 2015; Niedeggen et al., 2012; Niedeggen et al., 2002; Sahraie et al., 2001; Winther & Niedeggen, 2017b). This point has been addressed in previous studies (Hay et al., 2006). Notably, the design remains unimodal with respect to cue and target in all studies.

However, electrophysiological findings already suggested that the processing of the cue-target complex is different when cue and target were defined in distinct modalities (Kern & Niedeggen, 2021b): For unimodal DID (cue and target presented in the auditory domain), ERP responses to the cue-target compound were still characterized by a prominent P3 – if the target is detected successfully (Kern & Niedeggen, 2021a). Yet, correctly reported targets were additionally characterized by a stronger fronto-central negativity at about 200 ms. What is more, for the cross-modal DID effect (visual cue and auditory target), successful target detection was exclusively characterized by a pronounced fronto-central negativity, whereas the P3 amplitude was not enhanced when contrasted to misses (Kern & Niedeggen, 2021b).

These results suggests that the processing of the target is affected by the modality of the cue. From our point of view, this finding qualifies the task used in our study as a ‘cross-modal’ task.

We have clarified this point in our revised paper:

Page 5: “**In sum, these deviant ERP results signal that the central suppression process previously identified in uni-modal visual distractor studies must be questioned if cue and target are defined in different modalities.** **The processing of a target stimulus in a multiple-distractor task seems to be affected by the congruency of the cue’s modality. In this respect, we refer to studies in which cue and target are defined in the same modality as ‘unimodal’. The current study defines the cue and target in different sensory domains and is labeled ‘cross-modal’.”**

Please note that the ERP results of our study also emphasize the role of the different cue/target modalities: The posterior negativity – not observed in the uni-modal visual DIB studies (Niedeggen et al., 2015; Niedeggen et al., 2004; Niedeggen et al., 2012; Winther & Niedeggen, 2017a) – has probably been masked by the choice of the visual cue. Finally, the label ‘cross-modal’ is consistent with the labeling used in the previous studies – based on a similar setup (Kern & Niedeggen, 2021b, 2023).

Page 35: “**Replacing the visual cue by an auditory one reveals the discernibility of the VAN in the context of target processing. This suggests expanding the assumption that the cue merely serves as a temporal marker resolving attentional inhibition (Hay et al., 2006): The congruency of the cue with the target's modality appears to be an important experimental factor. To emphasize this fact, we decided to label the DIB effect associated with an auditory cue as a ‘cross-modal’ setup. Furthermore, this labeling is consistent with the labeling used in previous studies (Kern & Niedeggen, 2021b, 2023).**”

**Literature**

Arnell, K. M., & Shapiro, K. L. (2011). Attentional blink and repetition blindness. *Wiley interdisciplinary reviews. Cognitive science*, *2*(3), 336-344. <https://doi.org/10.1002/wcs.129>

Coltheart, V. (2010). A review of repetition blindness phenomena and theories. In *Tutorials in visual cognition.* (pp. 187-209). Psychology Press.

Greenhouse, S. W., & Geisser, S. (1959). On methods in the analysis of profile data. *Psychometrika*, *24*, 95-112. <https://doi.org/10.1007/BF02289823>

Hay, J. L., Milders, M. M., Sahraie, A., & Niedeggen, M. (2006). The effect of perceptual load on attention-induced motion blindness: The efficiency of selective inhibition. *Journal of Experimental Psychology: Human Perception and Performance*, *32*(4), 885-907. <https://doi.org/10.1037/0096-1523.32.4.885>

Kanwisher, N. G. (1987). Repetition blindness: Type recognition without token individuation. *Cognition*, *27*(2), 117-143. <https://doi.org/10.1016/0010-0277(87)90016-3>

Kern, L., & Niedeggen, M. (2021a). Distractor-induced deafness: The effect of multiple auditory distractors on conscious target processing. *Cortex*, *134*, 181-194. <https://doi.org/10.1016/j.cortex.2020.10.015>

Kern, L., & Niedeggen, M. (2021b). ERP signatures of auditory awareness in cross-modal distractor-induced deafness. *Consciousness and Cognition*, *96*, e103241. <https://doi.org/10.1016/j.concog.2021.103241>

Kern, L., & Niedeggen, M. (2023). Are auditory cues special? Evidence from cross-modal distractor-induced blindness. *Attention, Perception, & Psychophysics*, *85*(3), 889–904. <https://doi.org/10.3758/s13414-022-02540-0>

Michael, L., Hesselmann, G., Kiefer, M., & Niedeggen, M. (2011). Distractor-induced blindness for orientation changes and coherent motion. *Vision Research*, *51*(15), 1781-1787. <https://doi.org/10.1016/j.visres.2011.06.007>

Milders, M., Hay, J., Sahraie, A., & Niedeggen, M. (2004). Central inhibition ability modulates attention-induced motion blindness. *Cognition*, *94*(2), B23-B33. <https://doi.org/10.1016/j.cognition.2004.06.003>

Niedeggen, M., Busch, N. A., & Winther, G. N. (2015). The state of a central inhibition system predicts access to visual targets: An ERP study on distractor-induced blindness (DIB). *Consciousness and Cognition*, *35*, 308-318. <https://doi.org/10.1016/j.concog.2015.02.002>

Niedeggen, M., Hesselmann, G., Sahraie, A., Milders, M., & Blakemore, C. (2004). Probing the prerequisites for motion blindness. *Journal of cognitive neuroscience*, *16*(4), 584-597. <https://doi.org/10.1162/089892904323057317>

Niedeggen, M., Michael, L., & Hesselmann, G. (2012). Closing the gates to consciousness: distractors activate a central inhibition process. *Journal of cognitive neuroscience*, *24*(6), 1294-1304. <https://doi.org/10.1162/jocn_a_00177>

Niedeggen, M., Sahraie, A., Hesselmann, G., Milders, M., & Blakemore, C. (2002). Is experimental motion blindness due to sensory suppression? An ERP approach. *Cognitive Brain Research*, *13*(2), 241-247. <https://doi.org/10.1016/s0926-6410(01)00122-7>

Pinilla, T., Cobo, A., Torres, K., & Valdes-Sosa, M. (2001). Attentional shifts between surfaces: effects on detection and early brain potentials. *Vision Research*, *41*(13), 1619-1630. <https://doi.org/10.1016/s0042-6989(01)00039-6>

Rodríguez, V., & Valdés-Sosa, M. (2006). Sensory suppression during shifts of attention between surfaces in transparent motion. *Brain Research*, *1072*(1), 110-118. <https://doi.org/10.1016/j.brainres.2005.10.071>

Sahraie, A., Milders, M., & Niedeggen, M. (2001). Attention induced motion blindness. *Vision Research*, *41*(13), 1613-1617. <https://doi.org/10.1016/s0042-6989(01)00065-7>

Valdes-Sosa, M., Bobes, M. A., Rodriguez, V., & Pinilla, T. (1998). Switching attention without shifting the spotlight object-based attentional modulation of brain potentials. *Journal of cognitive neuroscience*, *10*(1), 137-151. <https://doi.org/10.1162/089892998563743>

Whittlesea, B. W. A., & Masson, M. E. J. (2005). Repetition Blindness in Rapid Lists: Activation and Inhibition Versus Construction and Attribution. *Journal of Experimental Psychology: Learning, Memory, and Cognition*, *31*(1), 54-67. <https://doi.org/10.1037/0278-7393.31.1.54>

Winther, G. N., & Niedeggen, M. (2017a). Adaptability and specificity of inhibition processes in distractor-induced blindness. *Psychophysiology*, *54*(12), 1882-1898. <https://doi.org/10.1111/psyp.12974>

Winther, G. N., & Niedeggen, M. (2017b). Distractor-Induced Blindness: A Special Case of Contingent Attentional Capture? *Advances in Cognitive Psychology*, *13*(1), 52-63. <https://doi.org/10.5709/acp-0206-5>

Winther, G. N., & Niedeggen, M. (2018). Differential activation of a central inhibition system by motion and colour distractors. *Visual Cognition*, *26*(10), 802-816. <https://doi.org/10.1080/13506285.2018.1561566>
