# Supplementary material for: Event-related potentials of stimuli inhibition and access in cross-modal distractor-induced blindness
Source: PLoS One. 2024 Oct 23;19(10):e0309425. doi: 10.1371/journal.pone.0309425 (PMC11498723; doi:10.1371/journal.pone.0309425)
Supplement: Attachment — Submitted filename: Response to Reviewers.docx [file pone.0309425.s009.docx]

Dear Dr. Patrick Bruns,

Please find enclosed again our revised manuscript (Event-related potentials of stimuli inhibition and access in cross-modal distractor-induced blindness) submitted for your consideration for publication in PLOS ONE.

Once again, we would like to express our gratitude to you and the second reviewer for the thoughtful remarks on our manuscript. We have thoroughly reviewed the feedback and made every effort to address the requirements.

In response to the reviewers' concerns about our use of the term ‘cross-modal’, we have expanded the limitations section of the revised manuscript to include a thorough discussion of the associated implications and constraints. We are confident that these detailed explanations will address the reviewer's feedback and substantiate the classification of our paradigm as 'cross-modal.' Additionally, we have conducted a further analysis that confirmed the re-test reliability of our results successfully.

The corresponding changes in the revised manuscript (see: Revised Manuscript with Track Changes) are detailed below, point by point. We hope that these substantial revisions and additions will now meet the reviewers' expectations and that the improved quality of the manuscript will be sufficient for publication in your esteemed journal.

Sincerely,

Sophie Hanke (on behalf of the authors)

**Reviewer #2:**

Once more, thank you for your detailed, valuable feedback. We have thoroughly reviewed your remarks and made every effort to address the requirements. Below you will find our responses, point by point. The parts in bold font are part of the revised manuscript, specified you find the corresponding page number.

*The reason the authors consider the effect being cross-modal is that the cue modality was found to affect target processing. In this case, what is cross modality should be "cue-induced target sensitivity" rather than the "distractor induced blindness". I still think that given the DIB phenomenon is centered on the effect of the distractor, the modality of interest should be between the distractor and the target, rather than the cue. This should, at the very least, be acknowledged as a limitation and discussed. The suboptimal operationalization in previous studies does not justify the continue of the conduct.*

*In the newly added contents, the authors mentioned several ERP components that only emerge in cross-modal DID experiments but not uni-modal experiments. One question is whether these effects are truly due to cross modality in general or caused by the particular modality of a *visual* stimuli in addition to auditory materials in Kern and Niedeggen (2023). The present study served as a nice control condition to the previous study by reversing the modality of the cue and target, creating a fully crossed modality manipulation when viewed together with the previous DID study. However, given that the findings of the present research suggest an auditory cue does not alter the visual DIB effect, can we conclude that the previous finding of a visual cue altering the auditory DID is an effect unique to the visual modality of the cue, rather than a general cross-modality effect?*

From a purely behavioral perspective, the DIB phenomenon appears consistent: Visual distractors seem to induce "blindness" towards a visual target, irrespective of the cue's modality, be it auditory or visual (Kern & Niedeggen, 2021b, 2023). However, the cue’s modality influences not only cue-target-evoked potentials but also distractor-evoked potentials. The statement that an auditory cue does not alter the visual DIB effect is true only on a behavioral level. The underlying neural processes involved do not appear to be identical - rather, they depend on the congruency of the cue-target modality.

Regarding the distractor-evoked potentials, the latency of the FN is shifted. In our experiment, we observed an onset latency of 90 ms, which is surprisingly early. In contrast, previous unimodal DIB studies have reported onset latencies between 225 and 325 ms (Niedeggen et al., 2015; Niedeggen et al., 2004; Niedeggen et al., 2012; Winther & Niedeggen, 2017). In our manuscript we cautiously attribute this shift in latency to the requirements of the cross-modal setting.

Page 34: “**In contrast to the previous unimodal DIB-studies, attentional resources in the visual domain can be focused on changes in the dynamic random dot pattern and are not to be ‘shared’ with the monitoring of a second visual stream including the transient cue. The distribution of attentional resources to two modalities might affect the processing speed in the visual domain.”**

What is more, the processing of cue-target-evoked ERPs, where the visual target and the auditory cue are processed simultaneously, appears to be entirely different. To illustrate this, we compare cue-target ERPs from an unimodal visual DIB experiment (see Figure 1, Niedeggen et al., 2015) with those from an auditory DID experiment (see Figure 2, Kern & Niedeggen, 2021a), as well as our current data from cross-modal DIB (see Figure 3).

**Figure 1**

Visual DIB

**Figure 2**

Auditory DID

**Figure 3**

Cross-modal DIB

During purely visual DIB (see Figure 1), target access appears to rely on late processing (P3), with no involvement of sensory processing. In contrast, during auditory DID (see Figure 2), target access depends on both early (FN) and late processing (P3). Hence, for unimodal investigations, late processing is consistently involved. However, when both modalities are combined in cross-modal DIB (see Figure 3), target access depends solely on early sensory processing (VAN), late processing no longer plays a role.

It is important to note that, at this time, we cannot determine if these two differing changes in the ERP signal are due to the general cross-modality of the effect. Distractor-evoked ERPs for cross-modal DID have not yet been investigated.

However, we advocate for retaining the label ‘cross-modal’ because we interpret our results, along with previous findings, as evidence for a significantly different processing when cue and target are defined in incongruent modalities. Nevertheless, as rightfully urged by the reviewer, we now discuss this point extensively in the limitations section of our revised manuscript.

Page 38: “**In the present study, the term 'cross-modal' specifically refers to the incongruent modality between target and cue, rather than target and distractors. Previous research has demonstrated that a crucial prerequisite for the DIB phenomenon to arise is the presence of distractors that share the visual features of the target (Michael et al., 2011; Michael et al., 2012). Given the significant differences in properties between auditory and visual stimuli, no blindness effect is expected to occur, if auditory distractors were paired with a visual target. Nevertheless, while in our setup blindness is triggered by visual distractors, the experimental task still requires simultaneous monitoring of two streams located in different modalities. Both streams contain stimuli necessary for successful task completion. With the cue and the target potentially appearing in parallel, it can be assumed that attention is divided between the two streams. What is more, our results indicate that monitoring the auditory stream in addition to the visual one seems to have an influence on the underlying neural processes detected in the EEG signal in response to the distractors as well as the target. However, whether these deviations, specifically regarding the distractor-evoked potentials, stem from a general effect of cross-modality, remains unclear. To elucidate this, it will be necessary to examine distractor-evoked potentials from the opposing cross-modal direction – in response to auditory distractors paired with an auditory target and a visual cue. While such results are still forthcoming, we preliminary advocate for using the label ‘cross-modal’ in the present study. Taken together, we interpret our results, along with previous findings, as evidence for a significantly different processing when cue and target are defined in incongruent modalities.**

**An alternative approach to multimodal research regarding the DIB/DID paradigm could involve equipping target and distractors with auditory as well as visual features at the same time. If the distractors share the target’s visual as well as auditory properties, a blindness/deafness effect is expected to occur. This method would emphasize the modality of interest between distractors and target in a more integrative manner, focusing on how shared features across modalities influence the phenomenon. However, research by Michael and colleagues (2012) from the visual domain suggests that, even in a combined-feature task, feature representations are expected to remain separate.**”

In addition, the cross-modal effect described by the authors--cue modality affects target identification--sounds like only a main effect, which is also likely well studied. Ideally, it would only make sense to vary cue validity in the current context if there has been initial evidence suggesting that cue validity modulates the effect of the distractors on target--an interaction between cue and distractor in DIB or DID, rather a simple main effect of the cue. I hope the authors make it clearer in the paper what kind of modulation effect the cue has.

First and foremost, we want to clarify that cue validity was not systematically manipulated as an independent variable in the present experiment, and thus, was not expected to have an effect on the electrophysiological results. Conditions in which the cue appeared without a target were included to ensure participant engagement and to filter out individuals who merely responded “yes” to all questions, regardless of what they actually saw or heard. These conditions were handled as control conditions. Therefore, conditions in which the cue did not reliably predict the target were substantially outnumbered (50 trials), compared to those where the target consistently followed the cue (140 trials).

The present experiment was designed solely to investigate distractor- as well as cue-target-evoked potentials in regards to visual distractor and target stimuli combined with an auditory cue. These ERPs were previously unknown. Consequently, our focus was primarily on whether and how the cue modality influences distractor and target processing in this specific scenario. Further research is needed to clarify the specific nature of the modulation effect observed. To achieve this, it would be beneficial to examine distractor-evoked potentials in the opposing cross-modal direction, as already mentioned above.

*My concern regarding the reliability given the small number of still holds. The authors mentioned that 55 trials were used per condition, and artifact rejection abandoned around 50% of the trials. (1) Please specify whether there were 55 trials before or after artifact rejection. If it is after rejection, only then less than 30 trials were available for analysis, which could be too few for reliable average waveforms. (2) It is unusual to see such a large proportion of trials abandoned in artifact rejections, making it questionable whether the remaining trials are truly representative of the overall performance. In other words, the effect may only occur for a selected subset of the trials after rejection. Please provide some clarification on why artifacts such as eye movements and head movements were not controlled during the experiment, but instead harshly rejected in later processing.*

Concerning the first point (1), we clarified in the revised manuscript that 55 trials per distractor condition were available for analysis prior to the artefact rejection process.

Page 11: **For the analysis of the distractor-evoked potentials, before artefact rejection, 55 trials were available for each distractor condition (1 vs. 3-4 vs. 5-6), with the SOA and control conditions combined. In previous studies, the analysis of distractor-evoked ERPs was based on a comparable number (n = 63 trials per distractor condition; Niedeggen et al., 2012; Winther & Niedeggen, 2017).**

It is correct to note that, on average, fewer than 30 trials were available for the final analysis. We already specified this in our original manuscript.

Page 14: **Per participant, the electrophysiological analysis of the probe state was finally based on a mean of 27.15 (SD = 6.73) out of 55 trials from the ‘single distractor’ condition, 27.00 (SD = 6.55) out of 55 trials from the ‘3-4 distractors’ condition, and 25.19 (SD = 7.55) out of 55 trials from the ‘5-6 distractors’ condition, after the artefact rejection process.**

We acknowledge the concern that these numbers may be insufficient for reliably averaging waveforms and take this doubt very seriously. To address this point and to account for the loss of trials – which reduces the signal-to-noise-ratio – we run an additional analysis of the distractor ERP, where the loss of trials was particularly pronounced.

First of all, we implemented a fully automated artefact rejection process, which controlled exclusively for eye blinks (vEOG channels >80 µV), transient over regulations (no signal for more than 100 ms), as well as alpha activity in the baseline range of the frontal electrodes (+40 to -40 µV between -100 and +100 ms). Artefacts at other electrodes and drifts – usually controlled for manually - were not considered. This approach led to a significant increase in trial numbers, resulting in an average of 42.96 trials (SD = 9.97) for the 'single distractor' condition, 41.48 trials (SD = 10.61) for the '3-4 distractors' condition, and 41.89 trials (SD = 9.11) for the '5-6 distractors' condition. The rejection rate was thereby reduced to 24%.

In a second step, we formed new averages, separately for the three distractor conditions, based on this liberal artefact correction. Our analysis showed that the reliability of the frontal ERP signal was still provided: A cross-correlation of the averages per participant resulted in a mean correlation coefficient of .68 (SD = .18) for the ‘single distractor’ condition, .69 (SD = .19) for the ‘3-4 distractors’ condition, and .69 (SD = .18) for the ‘5-6 distractors’ condition.

A statistical analysis of the new ERP signals at the frontal leads in the time range of interest (90-170 ms) affirmed an increase in negativity (see S2 Table and S3 Figure; also in the supplement). More importantly, the rmANOVA also confirmed a significant linear trend (F(1,26) = 8.11, p = .008, n_p_^2^ = .238), consistent with the previously analyzed data after the conservative artefact rejection.

**S2 Table**

*Mean amplitudes for the distractor-evoked ERP (Frontal Negativity, 90-170ms) after the liberal and conservative artefact rejection per distractor condition, including the corresponding number of trials they were based on.*

|  | Frontal Negativity (90-170 ms) | | | |
| --- | --- | --- | --- | --- |
| Condition | Liberal Artefact Rejection (24% rejection rate) | | Conservative Artefact Rejection (52% rejection rate) | |
| 1 distractor | M = .127 [-.14, .393] | avg based on M = 42.96 trials (SD = 9.97) | M = -.233 [-.66, .20] | avg based on M = 27.15 trials (SD = 6.73) |
| 3-4 distractors | M = -.195 [-.551, .161] | avg based on M = 41.48 trials (SD = 10.61) | M = -.337 [-.70, .03] | avg based on M = 27.00 trials (SD = 6.55) |
| 5-6 distractors | M = -.562 [-9.49, -.18] | avg based on M = 41.89 trials (SD = 9.11) | M = -.787 [-1.19, -.39] | avg based on M = 25.19 trials (SD = 7.55) |

*Note*. Mean (in μV) and the corresponding 95% confidence interval (CI) for the grand averaged (GA) waveforms. Number of trials remaining after each artifact rejection process (liberal vs. conservative) is indicated by the mean and corresponding standard deviation. While the liberal rejection process lead to a rejection rate of 24%, the conservative rejection lead to a rejection rate of 52%.

**S3 Figure**

*Graphical representation of the mean amplitudes for the distractor-evoked ERP (Frontal Negativity, 90-170ms) following both liberal and conservative artifact rejection, categorized by distractor condition.*

To conclude, our additional data suggests that a liberal artefact rejection (without baseline drift correction) yields reliable results (see cross-correlations) and, what’s more, also indicates a statistically significant effect (see rmANOVA). However, this automated correction does not effectively recognize baseline drifts, which impact the validity of the results. Although participants were instructed to move their heads as little as possible and a chin rest was used, movements (and therefore baseline drifts) were not entirely preventable due to the lengthy trials (5 seconds each). Hence, we still see a more conservative evaluation as necessary. We now refer in the revised manuscript to our additional data, which are also provided in the supplement and data repository (https://doi.org/10.17632/93nxgxntbk.1).

Please note, that this additional analysis was selectively focused on the frontal electrodes, and that drifts and artefacts at other electrodes were not considered. Therefore, we like to refer to this analysis as an additional analysis testing the reliability of the frontal ERP effect.

Page 14: “**Nevertheless, to test the reliability of our data, we conducted an additional analysis of the distractor ERP, where a loss of trials after artefact rejection was particularly pronounced. For this additional analysis we implemented a more liberal, fully automated rejection process, controlling exclusively for eye blinks (vEOG channels >80 µV), transient over regulations (no signal for more than 100 ms), and alpha activity in the baseline range of the frontal electrodes (+40 to -40 µV between -100 and +100 ms). Artefacts at other electrodes and drifts – usually controlled for manually - were not considered. The results of this additional analysis replicated our findings and can be found in the supplement (see supplement S2 Table and S3 Figure). The additional dataset is available in the open repository (**[**https://doi.org/10.17632/93nxgxntbk.1**](https://doi.org/10.17632/93nxgxntbk.1)**).**”

**Literature**

Kern, L., & Niedeggen, M. (2021a). Distractor-induced deafness: The effect of multiple auditory distractors on conscious target processing. *Cortex*, *134*, 181-194. <https://doi.org/10.1016/j.cortex.2020.10.015>

Kern, L., & Niedeggen, M. (2021b). ERP signatures of auditory awareness in cross-modal distractor-induced deafness. *Consciousness and Cognition*, *96*, e103241. <https://doi.org/10.1016/j.concog.2021.103241>

Kern, L., & Niedeggen, M. (2023). Are auditory cues special? Evidence from cross-modal distractor-induced blindness. *Attention, Perception, & Psychophysics*, *85*(3), 889–904. <https://doi.org/10.3758/s13414-022-02540-0>

Michael, L., Hesselmann, G., Kiefer, M., & Niedeggen, M. (2011). Distractor-induced blindness for orientation changes and coherent motion. *Vision Research*, *51*(15), 1781-1787. <https://doi.org/10.1016/j.visres.2011.06.007>

Michael, L., Kiefer, M., & Niedeggen, M. (2012). The influence of distracter and target features on distracter induced blindness. *Advances in Cognitive Psychology*, *8*(1), 62-69. <https://doi.org/10.2478/v10053-008-0103-3>

Niedeggen, M., Busch, N. A., & Winther, G. N. (2015). The state of a central inhibition system predicts access to visual targets: An ERP study on distractor-induced blindness (DIB). *Consciousness and Cognition*, *35*, 308-318. <https://doi.org/10.1016/j.concog.2015.02.002>

Niedeggen, M., Hesselmann, G., Sahraie, A., Milders, M., & Blakemore, C. (2004). Probing the prerequisites for motion blindness. *Journal of cognitive neuroscience*, *16*(4), 584-597. <https://doi.org/10.1162/089892904323057317>

Niedeggen, M., Michael, L., & Hesselmann, G. (2012). Closing the gates to consciousness: distractors activate a central inhibition process. *Journal of cognitive neuroscience*, *24*(6), 1294-1304. <https://doi.org/10.1162/jocn_a_00177>

Winther, G. N., & Niedeggen, M. (2017). Adaptability and specificity of inhibition processes in distractor-induced blindness. *Psychophysiology*, *54*(12), 1882-1898. <https://doi.org/10.1111/psyp.12974>
